# Metagenomes and Metagenome-Assembled Genomes from Microbiomes Metabolizing Thin Stillage from an Ethanol Biorefinery

**DOI:** 10.1128/mra.00290-22

**Published:** 2022-07-18

**Authors:** Nathaniel W. Fortney, Kevin S. Myers, Abel T. Ingle, Kevin A. Walters, Matthew J. Scarborough, Timothy J. Donohue, Daniel R. Noguera

**Affiliations:** a Great Lakes Bioenergy Research Center, University of Wisconsin-Madison, Madison, Wisconsin, USA; b Wisconsin Energy Institute, University of Wisconsin-Madison, Madison, Wisconsin, USA; c Department of Civil and Environmental Engineering, University of Wisconsin-Madison, Madison, Wisconsin, USA; d Department of Bacteriology, University of Wisconsin-Madison, Madison, Wisconsin, USA; e Department of Civil and Environmental Engineering, University of Vermont, Burlington, Vermont, USA; University of Maryland School of Medicine

## Abstract

Here, we report the metagenomes from five anaerobic bioreactors, operated under different conditions, that were fed carbohydrate-rich thin stillage from a corn starch ethanol plant. The putative functions of the abundant taxa identified here will inform future studies of microbial communities involved in valorizing this and other low-value agroindustrial residues.

## ANNOUNCEMENT

**FIG 1:**
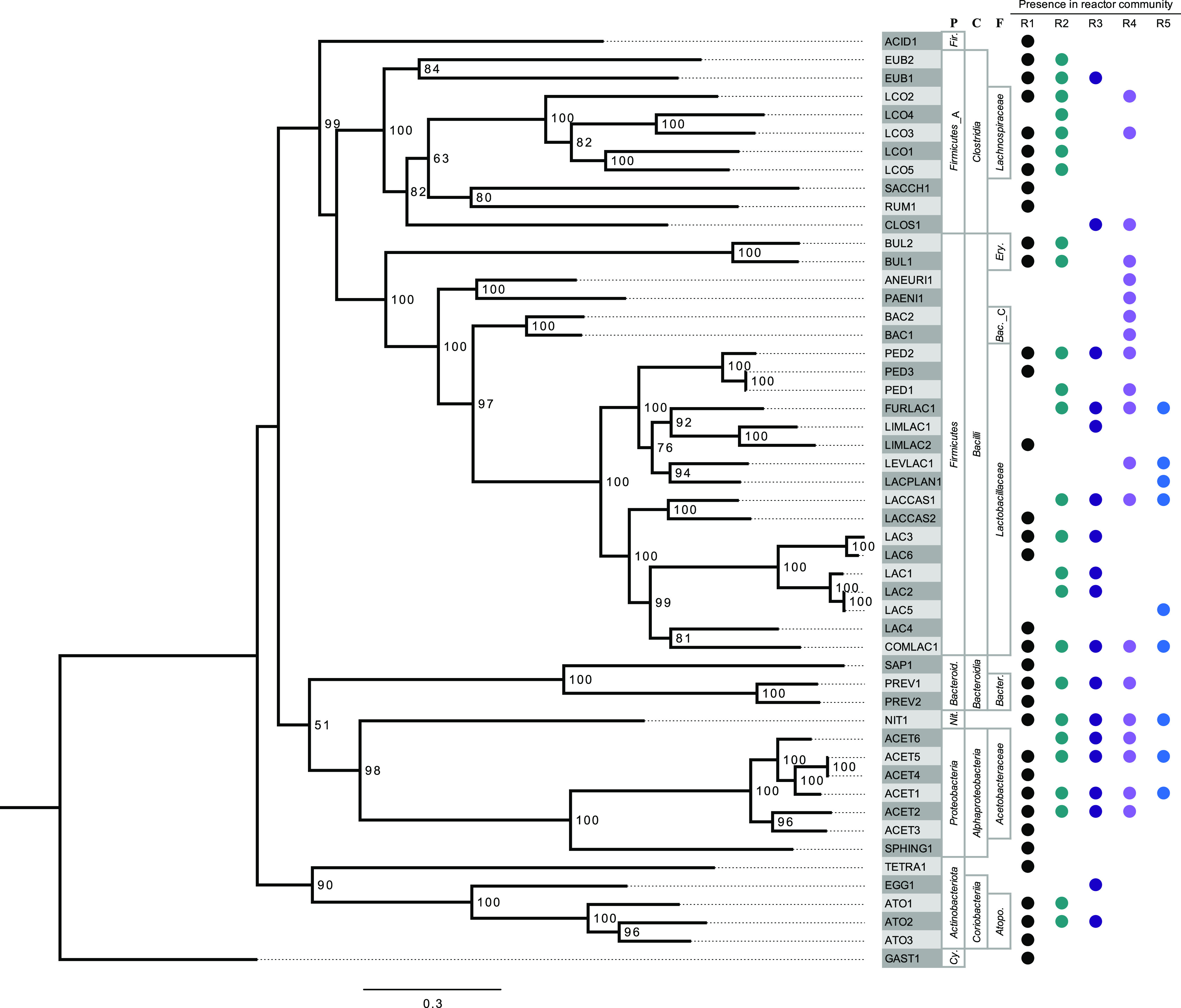
Phylogeny of representative MAGs and their presence in the five different bioreactors (R1 to R5), as determined by the dRep analysis (Table 1). R1, reactor 1, TS (R1_TS_); R2, reactor 2, solids-removed TS (R2_SR-TS_); R3, reactor 3, low SRT (R3_LowSRT_); R4, reactor 4, high temperature and low pH (R4_T-pH_); R5, reactor 5, low SRT, high temperature, and low pH (R5_T-pH-LowSRT_). The bioreactor operating conditions are described elsewhere (4). ACET, *Acetobacter*; ACID, *Acidaminococcus*; ANEURI, *Aneurinibacillus*; ATO, *Atopobiaceae*; BAC, *Bacillus*; BUL, *Bulleidia*; CLOS, *Clostridium*; COMLAC, *Companilactobacillus*; EGG, *Eggerthellaceae*; EUB, *Eubacteriaceae*; FURLAC, *Furfurilactobacillus*; GAST, *Gastranaerophilaceae*; LAC, *Lactobacillus*; LACCAS, *Lacticaseibacillus*; LACPLAN, *Lactiplantibacillus*; LCO, *Lachnospiraceae*; LEVLAC, *Levilactobacillus*; LIMLAC, *Limosilactobacillus*; NIT, *Nitrospira*; PAENI, *Paenibacillus*; PED, *Pediococcus*; PREV, *Prevotella*; RUM, *Ruminococcus*; SACCH, *Saccharofermentans*; SAP, *Saprospiraceae*; SPHING, *Sphingobium*; TETRA, *Tetrasphaera*. Higher taxonomic levels are labeled, from left to right, phylum (P), class (C), and family (F). *Fir.*, *Firmicutes_*C; *Bacteroid.*, *Bacteroidota*; *Nit*., *Nitrospirota*; *Cy.*, *Cyanobacteria*; *Ery*., *Erysipelotrichaceae*; *Bac._*C, *Bacillaceae*_C; *Bacter*., *Bacteroidaceae*; *Atopo.*, *Atopobiaceae*. The phylogenetic tree was generated in RAxML-ng with 500 bootstraps using the housekeeping gene concatenations generated by GTDB-tk, with the default selection of 120 single-copy bacterial housekeeping genes. Bootstrap values greater than 50 are shown. The scale bar indicates the number of nucleotide substitutions per sequence site.

We are investigating how to use anaerobic microbial communities for valorizing agroindustrial residues (1–5). We reported on fermentation products when thin stillage (TS) from starch bioethanol production was fed to a set of bioreactors (4). In that study, an anaerobic bioreactor (R1_TS_) was inoculated with acid-phase digester sludge from the Nine Springs Wastewater Treatment Plant (Madison, WI, USA) and provided TS as the feedstock. Four additional bioreactors (R2_SR-TS_, R3_LowSRT_, R4_T-pH_, and R5_T-pH-LowSRT_), derived from R1_TS_, were operated with different temperatures, pH values, and solids retention times (SRTs), resulting in diverging microbial communities and different fermentation products (4). Genomic DNA was extracted during bioreactor operation (R1_TS_, 6 samples; R2_SR-TS_, 9 samples; R3_LowSRT_, 6 samples; R4_T-pH_, 6 samples; R5_T-pH-LowSRT_, 2 samples) using a phenol-chloroform extraction method (2). DNA quantity and quality were determined using a Qubit 4 fluorometer (Thermo Fisher Scientific, USA) and NanoDrop ND-1000 spectrophotometer (Thermo Fisher Scientific), respectively. DNA aliquots of 500 ng (25 samples) and 3,000 ng (4 samples) were submitted to the Joint Genome Institute (JGI) for paired-end 2 × 150-bp NovaSeq S4 (Illumina, USA) and Sequel II (Pacific Biosciences [PacBio], USA) sequencing, respectively. Illumina library preparation followed established protocols (6). PacBio sequencing library preparation included shearing of genomic DNA (g-TUBE; Covaris, LLC, USA) to 6 to 10 kb and ligation using the SMRTbell Express template preparation 2.0 kit following the manufacturer’s protocol (PacBio). The resulting Illumina libraries contained between 70 million and 141 million 150-bp reads, and the PacBio libraries contained between 38 thousand and 159 thousand reads 6 to 9 kb in length. Illumina reads were filtered and error corrected using BBMap (v38.86) (mincount=2, highcountfraction=0.6) (7), assembled with metaSPAdes (v3.14.1) (8), and mapped with BBMap (v38.86) (ambiguous=random) (7) following the JGI Metagenomic Workflow (6). PacBio reads were filtered using BBtools (v38.87/38.88) (7), and CCS reads were assembled using metaFlye (v2.8.1-b1676) (3), polished with subreads using GCpp (v1.0.0-SL-release-8.0.0) (https://github.com/PacificBiosciences/gcpp), mapped using minimap2 (v2.17-r941) (4), and then binned with MetaBAT (v2:2.15) (9). The resulting metagenome-assembled genomes (MAGs) were refined by removing contigs deemed to be contaminants by ProDeGe (v2.3) (10) and a custom algorithm that compares tetranucleotide frequency among contigs (run.GC.sh and Calculating_TF_Correlations.R [https://github.com/GLBRC/metagenome_analysis]). The MAGs obtained from individual samples were dereplicated using dRep (v3.2.2) (dereplicate command with –conW 0.5 and –N50W 5 flags for custom weighting) (11). MAG quality parameters were obtained using CheckM (v1.0.11) (12), and taxonomy was assigned using GTDB-tk (v1.5.1, database release 202) (13). MAG phylogeny was visualized using RAxML-NG (v0.9.0) (Fig. 1) (14). MAGs were annotated through the NCBI Prokaryotic Genome Annotation Pipeline (PGAP) (15).

We report a total of 266 MAGs with >75% completion, grouped in 51 clusters that represent the diversity in the individual bioreactors (Table 1). This metagenomic data set adds to the expanding body of knowledge about microorganisms relevant to the valorization of agroindustrial residues by fermentation (2, 16–20).

**TABLE 1:** Genome accession numbers and statistics

Strain name^*a*^	Code^*b*^	Reactor source^*c*^					Sample age (days)	SRA accession no.	No. of raw reads per sample (×1,000)	GenBank accession no.^*d*^	ANIm^*e*^	dRep^*f*^	GTDBtk classification	Reference genome^*g*^	Sequencing platform^*h*^	Completeness (%)	Contamination (%)	MAG size (Mbp)	No. of scaffolds	*N*_50_ (Mbp)	GC content (%)	No. of tRNAs	No. of 5S rRNAs	No. of 16S rRNAs	No. of 23S rRNAs
		R1	R2	R3	R4	R5																			
UW_TS_ACET1_1	ACET1		X				100	SRX12729178	383	JAKVNI000000000		132.1938	d__Bacteria;p__Proteobacteria;c__Alphaproteobacteria;o__Acetobacterales;f__Acetobacteraceae;g__Acetobacter;s__Acetobacter fabarum	GCF_011516925.1	Sequel II	100	0.25	2.896	1	2.896	58.3	59	5	5	5
UW_TS_ACET1_2					X		72	SRX12687768	95,453	JALCKF000000000	0.999992	123.6701			NovaSeq S4	98.51	0.25	2.37	25	0.138	58.8	39	0	0	0
UW_TS_ACET1_3				X			24	SRX12665963	80,367	JALCHP000000000	0.999833	123.6696			NovaSeq S4	98.51	0.25	2.271	24	0.138	59.1	40	0	0	0
UW_TS_ACET1_4			X				136	SRX12664702	125,596	JALCGX000000000	0.999986	123.5262			NovaSeq S4	99	0.25	2.392	26	0.102	58.8	41	0	0	0
UW_TS_ACET1_5					X		13	SRX12687755	74,621	JALCJH000000000	0.999995	123.1801			NovaSeq S4	98.01	0.25	2.211	24	0.138	59.1	38	0	0	0
UW_TS_ACET1_6					X		24	SRX12686847	69,380	JALCJQ000000000	0.999991	123.1801			NovaSeq S4	98.01	0.25	2.211	24	0.138	59.1	37	0	0	0
UW_TS_ACET1_7				X			66	SRX12686873	104,400	JALCIB000000000	0.999984	123.1801			NovaSeq S4	98.01	0.25	2.212	24	0.138	59.1	37	0	0	0
UW_TS_ACET1_8				X			79	SRX12686849	79,309	JALCII000000000	0.999983	123.1801			NovaSeq S4	98.01	0.25	2.212	23	0.138	59.1	37	0	0	0
UW_TS_ACET1_9					X		162	SRX12687754	66,381	JALCKU000000000	0.999983	123.1801			NovaSeq S4	98.01	0.25	2.225	24	0.138	59.1	37	0	0	0
UW_TS_ACET1_10				X			48	SRX12670963	74,191	JALCHU000000000	0.999983	123.1801			NovaSeq S4	98.01	0.25	2.212	22	0.138	59.1	37	0	0	0
UW_TS_ACET1_11			X				64	SRX12658907	90,171	JALCFX000000000	0.999994	123.1801			NovaSeq S4	98.01	0.25	2.227	23	0.138	59.1	37	0	0	0
UW_TS_ACET1_12				X			162	SRX12686848	92,161	JALCIX000000000	0.999971	123.1801			NovaSeq S4	98.01	0.25	2.213	22	0.138	59.1	38	0	0	0
UW_TS_ACET1_13						X	36	SRX12729153	71,598	JAKVLI000000000	0.999989	123.1800			NovaSeq S4	98.01	0.25	2.225	24	0.138	59.1	38	0	0	0
UW_TS_ACET1_14						X	66	SRX12729463	81,580	JAKVLD000000000	0.999974	123.1800			NovaSeq S4	98.01	0.25	2.226	24	0.138	59.1	38	0	0	0
UW_TS_ACET1_15			X				202	SRX12667031	121,391	JALCHJ000000000	0.999985	123.0262			NovaSeq S4	98.51	0.25	2.304	24	0.102	59	39	0	0	0
UW_TS_ACET1_16					X		62	SRX12687723	84,579	JALCJZ000000000	0.999985	122.1801			NovaSeq S4	97.01	0.25	2.182	23	0.138	59.2	36	0	0	0
UW_TS_ACET1_17		X					39	SRX12657440	113,675	JALCEB000000000	0.999964	120.1678			NovaSeq S4	95.52	0.75	2.27	44	0.102	59.1	41	0	0	0
UW_TS_ACET1_18					X		120	SRX12687759	78,913	JALCKN000000000	0.999977	116.7101			NovaSeq S4	91.29	0.25	2.182	23	0.138	59.1	35	0	0	0
UW_TS_ACET1_19			X				90	SRX12660021	140,410	JALCGK000000000	0.999986	116.2101			NovaSeq S4	91.04	0.25	2.31	25	0.138	58.7	38	0	0	0
UW_TS_ACET1_20		X					63	SRX12729174	1,591	JAKVMC000000000	0.999976	112.8698			Sequel II	82.09	0.25	2.268	2	1.51	58.2	38	2	2	2
UW_TS_ACET1_21			X				52	SRX12729156	634	JAKVLO000000000	0.999996	112.8689			Sequel II	82.09	0.25	2.255	2	1.509	58.2	38	2	2	2
UW_TS_ACET1_22			X				166	SRX12729462	793	JAKVMT000000000	0.999093	107.3584			Sequel II	82.59	0.25	2.086	21	0.115	58.7	30	0	1	0
UW_TS_ACET1_23		X					75	SRX12658615	141,269	JALCEP000000000	0.999838	100.6800			NovaSeq S4	77.79	0.25	2.174	67	0.043	58.6	35	0	0	0
UW_TS_ACET2_1	ACET2		X				100	SRX12729178	383	JAKVNJ000000000		132.0261	d__Bacteria;p__Proteobacteria;c__Alphaproteobacteria;o__Acetobacterales;f__Acetobacteraceae;g__Acetobacter;s__Acetobacter sp012517935	GCA_012517935.1	Sequel II	100	1.24	3.367	1	3.367	54.4	60	5	5	5
UW_TS_ACET2_2			X				166	SRX12729462	793	JAKVMU000000000	0.999967	130.6313			Sequel II	100	1.24	3.423	3	1.771	54.4	60	5	5	5
UW_TS_ACET2_3		X					63	SRX12729174	1,591	JAKVMD000000000	0.999854	126.9446			Sequel II	95.02	1.24	3.213	1	3.213	54.3	51	4	4	4
UW_TS_ACET2_4					X		62	SRX12687723	84,579	JALCJY000000000	0.999998	125.3394			NovaSeq S4	100	0.25	3.014	30	0.123	53.9	45	0	0	0
UW_TS_ACET2_5		X					75	SRX12658615	141,269	JALCEO000000000	0.999995	125.2687			NovaSeq S4	100	0.25	2.931	29	0.119	53.9	45	0	0	0
UW_TS_ACET2_6					X		24	SRX12686847	69,380	JALCJP000000000	0.999860	125.2687			NovaSeq S4	100	0.25	3.058	30	0.119	53.9	45	0	0	0
UW_TS_ACET2_7			X				90	SRX12660021	140,410	JALCGJ000000000	0.999999	125.2685			NovaSeq S4	100	0.25	2.917	31	0.119	53.9	46	0	0	0
UW_TS_ACET2_8				X			114	SRX12686875	69,693	JALCIN000000000	0.999939	125.1004			NovaSeq S4	100	0.25	3.051	37	0.11	53.9	45	0	0	0
UW_TS_ACET2_9			X				136	SRX12664702	125,596	JALCGW000000000	0.999995	124.7733			NovaSeq S4	100	1.24	2.906	31	0.119	53.8	45	0	0	0
UW_TS_ACET2_10		X					39	SRX12657440	113,675	JALCEA000000000	0.999993	124.6916			NovaSeq S4	100	1.24	2.952	35	0.115	53.8	45	0	0	0
UW_TS_ACET2_11					X		120	SRX12687759	78,913	JALCKM000000000	0.999992	124.5971			NovaSeq S4	100	1.24	3.078	33	0.11	53.9	46	0	0	0
UW_TS_ACET2_12			X				28	SRX12658904	101,250	JALCFM000000000	0.999922	124.5573			NovaSeq S4	100	1.24	3.06	37	0.108	53.8	46	0	0	0
UW_TS_ACET2_13				X			48	SRX12670963	74,191	JALCHT000000000	0.999995	114.4437			NovaSeq S4	89.05	0	2.664	28	0.119	54	38	0	0	0
UW_TS_ACET2_14				X			79	SRX12686849	79,309	JALCIH000000000	0.999948	109.3823			NovaSeq S4	84.33	0.25	2.55	35	0.108	53.8	41	0	0	0
UW_TS_ACET3_1	ACET3	X					63	SRX12729174	1,591	JAKVME000000000		131.3647	d__Bacteria;p__Proteobacteria;c__Alphaproteobacteria;o__Acetobacterales;f__Acetobacteraceae;g__Acetobacter; s__	NA	Sequel II	99.75	0	3.708	3	2.636	57.6	53	3	3	3
UW_TS_ACET3_2		X					39	SRX12657440	113,675	JALCDZ000000000	0.999930	125.5032			NovaSeq S4	99	0	3.503	27	0.249	57.6	46	0	0	0
UW_TS_ACET3_3		X					75	SRX12658615	141,269	JALCEN000000000	0.999949	125.3846			NovaSeq S4	99	0	3.445	27	0.236	57.7	46	0	0	0
UW_TS_ACET4_1	ACET4	X					63	SRX12729174	1,591	JAKVMF000000000		130.1843	d__Bacteria;p__Proteobacteria;c__Alphaproteobacteria;o__Acetobacterales;f__Acetobacteraceae;g__Acetobacter;s__Acetobacter peroxydans	GCF_006539345.1	Sequel II	99.5	0.5	2.642	2	2.162	60.3	56	4	4	4
UW_TS_ACET5_1	ACET5		X				90	SRX12660021	140,410	JALCGI000000000		125.3562	d__Bacteria;p__Proteobacteria;c__Alphaproteobacteria;o__Acetobacterales;f__Acetobacteraceae;g__Acetobacter;s__Acetobacter peroxydans	GCF_006539345.1	NovaSeq S4	100	0	2.559	29	0.117	60.7	45	0	0	0
UW_TS_ACET5_2					X		13	SRX12687755	74,621	JALCJG000000000	0.999990	125.0534			NovaSeq S4	100	0	2.521	34	0.102	60.9	44	0	0	0
UW_TS_ACET5_3					X		162	SRX12687754	66,381	JALCKT000000000	0.999986	123.9439			NovaSeq S4	99.5	0	2.52	43	0.077	60.8	45	0	0	0
UW_TS_ACET5_4						X	66	SRX12729463	81,580	JAKVLE000000000	0.999982	122.0555			NovaSeq S4	97.71	0	2.499	46	0.074	60.9	42	1	0	1
UW_TS_ACET5_5					X		120	SRX12687759	78,913	JALCKL000000000	0.999971	117.5738			NovaSeq S4	94.35	0.5	2.272	72	0.039	61.1	45	0	0	0
UW_TS_ACET5_6			X				64	SRX12658907	90,171	JALCFW000000000	0.999948	117.2484			NovaSeq S4	94.5	0.17	2.213	83	0.037	61.2	41	0	0	0
UW_TS_ACET5_7					X		72	SRX12687768	95,453	JALCKE000000000	1.000000	102.3029			NovaSeq S4	76.12	0	1.815	15	0.172	60.8	31	0	0	0
UW_TS_ACET5_8			X				136	SRX12664702	125,596	JALCGV000000000	0.999979	99.8305			NovaSeq S4	77.59	0	2.185	89	0.028	61.1	36	1	0	1
UW_TS_ACET6_1	ACET6				X		120	SRX12687759	78,913	JALCKK000000000		114.6918	d__Bacteria;p__Proteobacteria;c__Alphaproteobacteria;o__Acetobacterales;f__Acetobacteraceae;g__Acetobacter;s__Acetobacter indonesiensis	GCF_000963945.1	NovaSeq S4	89.05	0	2.337	23	0.134	55.1	40	0	0	0
UW_TS_ACET6_2				X			162	SRX12686848	92,161	JALCIW000000000	0.999994	114.6918			NovaSeq S4	89.05	0	2.346	25	0.134	55.1	40	0	0	0
UW_TS_ACET6_3			X				136	SRX12664702	125,596	JALCGU000000000	0.999984	111.8759			NovaSeq S4	87.48	0.25	2.267	50	0.08	55.2	39	0	0	0
UW_TS_ACID1_1	ACID1	X					63	SRX12729174	15,91	JAKVMG000000000		129.8131	d__Bacteria;p__Firmicutes_C;c__Negativicutes;o__Acidaminococcales;f__Acidaminococcaceae;g__Acidaminococcus;s__Acidaminococcus provencensis	GCF_900291475.1	Sequel II	99.38	1.2	2.915	1	2.915	52.8	56	6	6	6
UW_TS_ANEURI1_1	ANEURI1				X		162	SRX12687754	66,381	JALCKS000000000		122.1157	d__Bacteria;p__Firmicutes;c__Bacilli;o__Aneurinibacillales;f__Aneurinibacillaceae;g__Aneurinibacillus;s__Aneurinibacillus aneurinilyticus	GCF_000466385.1	NovaSeq S4	99.22	1.68	5.042	94	0.088	43.6	85	0	3	6
UW_TS_ATO1_1	ATO1		X				166	SRX12729462	793	JAKVMV000000000		131.2388	d__Bacteria;p__Actinobacteriota;c__Coriobacteriia;o__Coriobacteriales;f__Atopobiaceae;g__CADBMC01;s__CADBMC01 sp902795635	GCA_902795635.1	Sequel II	99.85	0	1.888	1	1.888	67.4	45	2	2	2
UW_TS_ATO1_2			X				202	SRX12667031	121,391	JALCHI000000000	0.999804	129.0235			NovaSeq S4	100	0	1.864	4	0.635	67.5	45	0	0	0
UW_TS_ATO1_3			X				136	SRX12664702	125,596	JALCGT000000000	0.999804	128.9496			NovaSeq S4	100	0	1.861	5	0.614	67.5	45	0	0	0

### Data availability.

Raw metagenomic sequence data and MAGs are available in NCBI GenBank under BioProject accession number PRJNA768492. All information on library construction and sequence can be found at https://gold.jgi.doe.gov/study?id=Gs0150020 using JGI GOLD Study identification number Gs0150020. All custom scripts are available at GitHub (https://github.com/GLBRC/metagenome_analysis).
